# Using virtual global landmark to improve incidental spatial learning

**DOI:** 10.1038/s41598-022-10855-z

**Published:** 2022-04-25

**Authors:** Jia Liu, Avinash Kumar Singh, Chin-Teng Lin

**Affiliations:** Australian Artificial Intelligence Institute, School of Computer Science, Faculty of Engineering and Information Technology, University of Technology Sydney, Sydney, Australia

**Keywords:** Learning and memory, Perception, Attention

## Abstract

To reduce the decline of spatial cognitive skills caused by the increasing use of automated GPS navigation, the virtual global landmark (VGL) system is proposed to help people naturally improve their sense of direction. Designed to accompany a heads-up navigation system, VGL system constantly displays silhouette of global landmarks in the navigator’s vision as a notable frame of reference. This study exams how VGL system impacts incidental spatial learning, i.e., subconscious spatial knowledge acquisition. We asked 55 participants to explore a virtual environment and then draw a map of what they had explored while capturing electroencephalogram (EEG) signals and eye activity. The results suggest that, with the VGL system, participants paid more attention during exploration and performed significantly better at the map drawing task—a result that indicates substantially improved incidental spatial learning. This finding might kickstart a redesigning navigation aids, to teach users to learn a route rather than simply showing them the way.

## Introduction

Spatial abilities are considered to be amongst the most important capacities for the survival of all species^1–3^. Developing navigational skills is not only a benefit for independence and personal well-being but also has positive effects on the human brain^4–8^. The discovery of brain’s ‘global positioning system’ by O’Keefe’s team^7^ built up a direct bridge between our brain and orientation skills, which reveals that the ability to navigate could be associated with improved brain functions. This close relationship can be found when new mental maps are formed through the act of navigation, the neural connections then increase, and new neural pathways are created accordingly^4–8^. In other words, our navigation skills, i.e., our spatial learning ability, directly and positively affects the physical condition of our brain. A well-known study on the enlarged hippocampi of London cab drivers is a good example of how spatial navigation skills can biologically impact the brain’s cortical plasticity^9^. This is because during their working time, they are required to continuously process the surrounding streets and environment, which, in turn, expands their brain to accommodate the cognitive demands of navigating London’s streets.

Today, with the development of technology on the global positioning system (GPS), the essential demands of spatial navigation and orientation are being offloaded to automatic orientation systems. This technology undoubtedly benefits human navigation with reliable, efficient directions, especially in outdoor environments. However, as a negative effect, this automated support replaces our fundamental tendency to build up a cognitive map of our surroundings through self-active exploration with trials and errors^1,7,10^, in other words, it may turn off the brain’s own GPS. Following simple turn-by-turn navigation instructions can easily cause an ignorance of processing of environmental information^1,11,12^. Researchers have noted that people who use GPS-based navigation systems have a poorer sense of direction, which leads to a decline in spatial memory^11,13^. This declines in spatial memory can negatively impact the ability to construct mental maps^11–13^. One could argue that automated navigation services are damaging the development of spatial skills in humans^1,14,15^.

To overcome this spatial deskilling, a new generation of navigation assistance systems is being developed that incorporates some of the natural elements of the human internal navigation system. The idea is to incorporate more of one’s surroundings into the directions to positively influence spatial awareness in the brain^16,17^. More spatial cues while a person is moving through an environment might encourage the user to process more spatial information, which can help them retain more awareness and attention on their actual surroundings, even in unfamiliar territory. As one of the most basic external references for an environment, landmarks are easily recognized and remembered to serve as the key navigation cues^18,19^. Recent empirical studies have uncovered the benefits of associating landmarks with navigation instructions^16,20^. For instance, integrating references to landmarks into turn-by-turn verbal directions, e.g., “Please turn right at the concert hall.”, has proven to bolster spatial learning^1,16,21^. Similarly, displaying distant landmarks at the edge of a mobile screen to support spatial orientation positively contributes to the user’s wayfinding efficiency and awareness of spatial information for surrondings^20^. This approach may enhance how a user encodes spatial knowledge while using a navigation system. Overall, as a common heading reference, the application of landmarks on navigational aids could help to overcome the negative effects of relying on navigation assistance systems.

**Figure 1:**
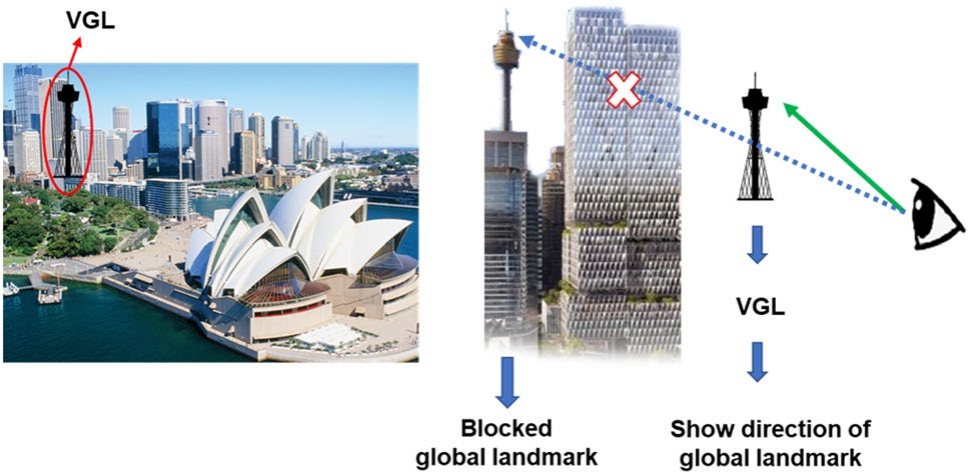
Working mechanism of the VGL system. When the selected global landmark reference is obscured from view, the VGL is displayed to constantly indicate its direction.

Inspired by the constructive role landmarks can play in spatial learning, we developed a system called virtual global landmark (VGL) system that incorporates global landmarks into a virtual heads-up display (see Fig. 1). The way the system works is that it constantly ensures a global landmark is visible, even when obscured by one’s surroundings. Global landmarks include things like city skylines or mountains—features that are visible from far away and serve as a broad frame of reference. In a process called landmark encoding, these landmarks do not disappear or change when the observer moves a small distance away^22^. Our idea is to ensure that the navigator is always aware of their own position relative to a global landmark. Learning this information not only helps people to develop a cognitive map of the environment, it is also considered to be one of the most significant strategies for the allocation of attention^23^ and developing spatial memory^7,10^. It therefore serves as a sort of proxy for a compass. Continuous neural processing of this basic point of reference could then help to increase route knowledge in an environment and in the meantime, grab more chance to motivate the user to continually survey the spatial knowledge, which contributes to spatial learning.

In this study, we examined the effects of the VGL system on spatial learning and, in particular, its impact on spatial knowledge acquisition. According to the model introduced by Siegel and White^24^, spatial knowledge acquisition can be described at different levels from the simple to the complex. The model also describes different types of spatial learning acquisition—landmark knowledge, which represents knowledge about objects in space; route knowledge, which refers to the connections between those landmarks in space; and survey knowledge, which is map-like knowledge that includes spatial relations between landmarks. Survey knowledge also enables complex computations like calculating short-cuts from previously unknown routes. Thus, to assess overall levels of spatial knowledge acquisition gained via the VGL system, we conducted a trial where the participants had to draw a map of a relatively unfamiliar environment. A map drawing task is a common method of evaluating the spatial knowledge gained from an environment that contains certain spatial information because it is robust to reflecting cognitive impact^25–27^.

A cohort of 55 students from the University of Technology Sydney participated in the study. Each was equipped with a head-mounted VR system, which they used to explore a medium-scale VR environment called “Sydney Park”, and a mobile brain/body imaging (MoBI)^28–30^ setup that captured brain dynamics via EEG signals while they explored. During exploration, a fixed route was predefined to control and standardize how participants explored the environment. The route was defined in a way that balanced the participant’s exposure to local and global landmarks. In this stage, the participants were divided into two groups for exploration. One group explored Sydney Park following auditory instructions, but with the VGL system equipped. The other did not use the VGL system; they simply followed the auditory instructions. The idea with providing the instructions in verbal form was to guide participants walked through the predefined route, as well as simulate navigation aids that provide turn-by-turn instructions. Adding the VGL system provided a way to assess an incidental knowledge acquisition alongside this guided navigation process. Before and during their explorations, the participants were not informed that they would subsequently be asked to draw a map of the route they had taken. We measured the fit of the sketch map’s configuration to the actual target configuration as a score against seven points of fidelity to show the outcome of spatial knowledge acquisition.

To investigate human brain spectral activity during exploration, we evaluated event-related spectral perturbations (ERSP) originating in the occipital and parietal cortices of the participants. The ERSP^31,32^ measured the average dynamic changes in amplitude of the broad band EEG frequency spectrum as a function of time relative to fixation events generated by the eye tracker. Many studies have demonstrated involvement by the parietal cortex in spatial navigation^33–36^. We then focused on brain activity in parietal regions to evaluate the brain encoding on the spatial knowledge related to navigation in exploration. And, as the visual processing area of our brains, the occipital lobe is responsible for interpreting the visual world around the body, such as the shape, color, and location of an object^37,38^. Thus, we were also interested to see whether brain dynamics in the occipital cortical areas would differ when participants were fixating on VGL as compared on other landmark stimuli during the exploration. We also took notes on behavioral activity and asked each of the participants to complete a Santa Barbara Sense of Direction^39^ (SBSOD) test together with Perspective Taking/Spatial Orientation Task^40^ (PTSOT) before completing the trials to control for individual differences in spatial abilities.

Based on our design of VGL system as a steady reference frame for surroundings, we hypothesized that participants using the VGL system during exploration would be more aware of the surroundings, process more spatial knowledge incidentally. Thus, in exploration phase, as measures to show whether more awareness and attention occur, we expected to find participants voluntary to spent longer time to complete the exploration and fixated longer in the environment. Being a method to visualize the outcomes of spatial knowledge processing in exploration, we expected to see an improved performance in map drawing task by participants with aid of VGL system, which would be the evidence of an increased spatial knowledge processed from exploration. Additionally, the mental demand investigated by NASA Task Load Index (NASA-TLX)^41^ was expected to show no increase for participants drawing with VGL information. Lastly, the increased alpha and decreased theta power over parietal and occipital cortex has been revealed by the suppressing distractions from the visual system^42–45^. Hence, to assess whether VGL could help attracting awareness and attention of the surroundings, we also expected to find the same phenomena in the ERSPs related to VGL stimuli around parietal and occipital sites.

## Results

Learning performance in the exploration and map drawing task were assessed via one-way ANOVAs. Separate ANOVAs were computed for: (i) total time spent in exploration; (ii) average blink intervals during exploration; (iii) NASA-TLX mental demand scores during the map drawing task; (iv) total map scores for the map drawing task; and (v) single indexes scores for the map drawing task, including canonical organization scores, recalled points percentage, rotational bias, scaling bias, canonical accuracy, distance accuracy, and angle accuracy. The group conditions, VGL and (non-) NVGL, were entered as a between-subjects factor. All the above indexes for both groups were normally distributed (*p* > 0.05). The Spearman’s correlations result between each individual’s spatial ability and the above indexes are shown in Fig. S1 of the supplementary. Significantly correlated factors were entered as covariates to compute the ANOVAs. Additionally, gender was added as a between-subjects factor when calculating one-way ANOVAs to evaluate gender effect on results. However, there was no significant interaction between gender and group condition for any of the measures. The test results of the between-subject effects are provided in the supplementary materials.

### VGL system users paid more attention during exploration

**Figure 2:**
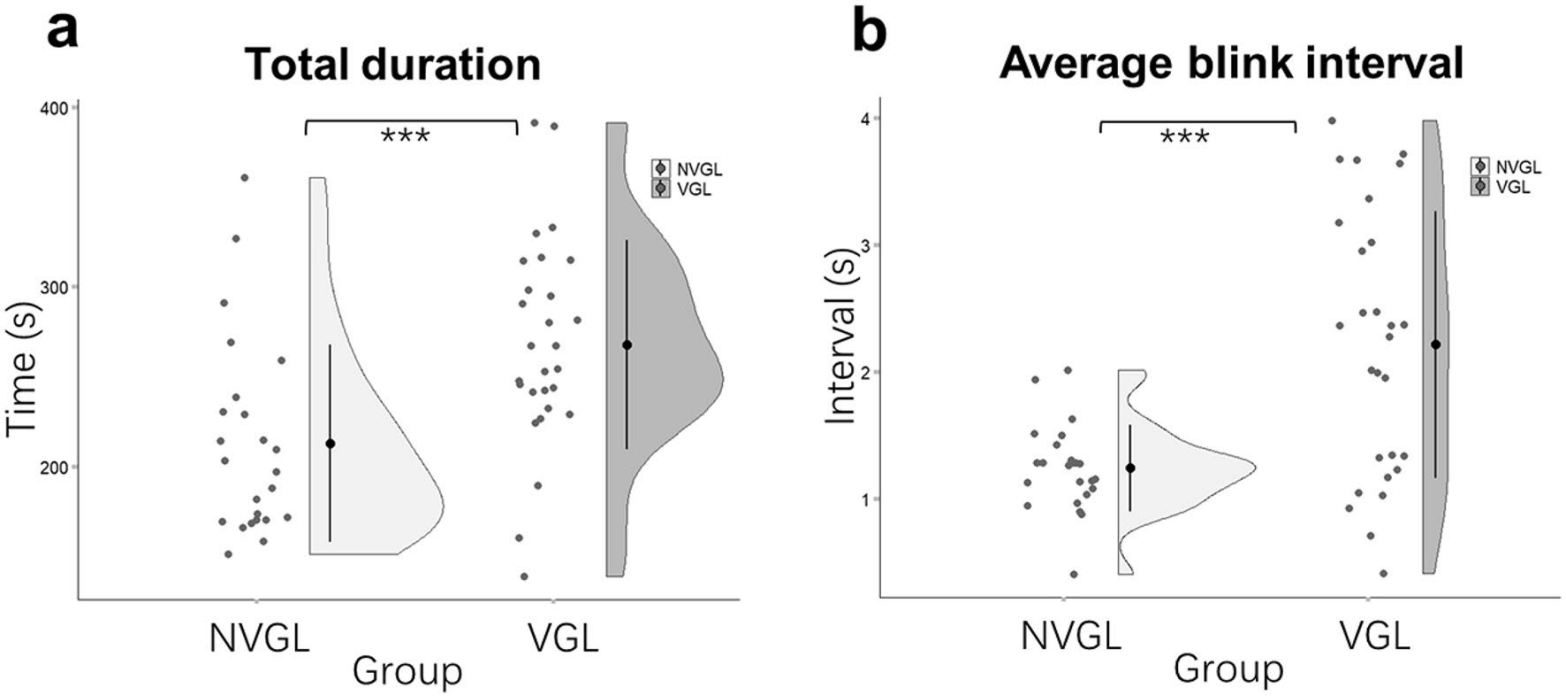
Comparisons between the VGL and NVGL groups in exploration. Each dot of the scatter plot indicates the average value for one participant. **(a)** Total time spent; **(b)** Average blink interval. The black dots represent means, and the error bars indicate standard deviations. *: *p* < .05 and ***: *p* < .001.

During the exploration phase of the trial, the participants determined their own walking speed. Hence, the time spent by the two groups was dependent on each participant. As presented in Fig. 2a, we found a statistically significant difference in the time spent (F_1,50_ = 12.08, *p* = 0.001, partial η^2^ = 0.20) with 54.83 s longer spent by the VGL group (M = 267.78, SE = 10.72) than the NVGL group (M = 212.95, SE = 11.58). To assess the impact of accuracy in navigation and response to verbal prompts on time spent during exploration, we analyzed the Spearman’s correlation between time spent and the number of wrong response to verbal prompts. The result reveals no significant correlation between these two (rs(55) = 0.16, p = 0.25). These findings suggest that participants aided by the VGL system walked more slowly and might be attracted more attention with significant more time spending while exploring. Additionally, due to the significant difference in time spent during exploration between two groups, we used this time for exploration as a covariate to compute ANOVAs for other measured indexes to control the time effect.

To find more evidence on attention during exploration, we also computed the average blink interval for each participant. As shown in Fig. 2b, the results are statistically significant (F_1,48_ = 14.02, *p* < 0.000, partial η^2^ = 0.23). The VGL group had a 0.86 s longer blink interval (M = 2.16, SE = 0.15) than the NVGL group (M = 1.30, SE = 0.16). The longer interval between blinks relates to a lower blink rate. By the interpretation of split attention revealed with higher blink rate^46,47^, the significant longer blink interval for VGL group indicates an increased attention while exploring when participants were aided by VGLs.

### VGL system users scored higher for incidental spatial knowledge acquisition in the map drawing task without additional mental workload

**Figure 3:**
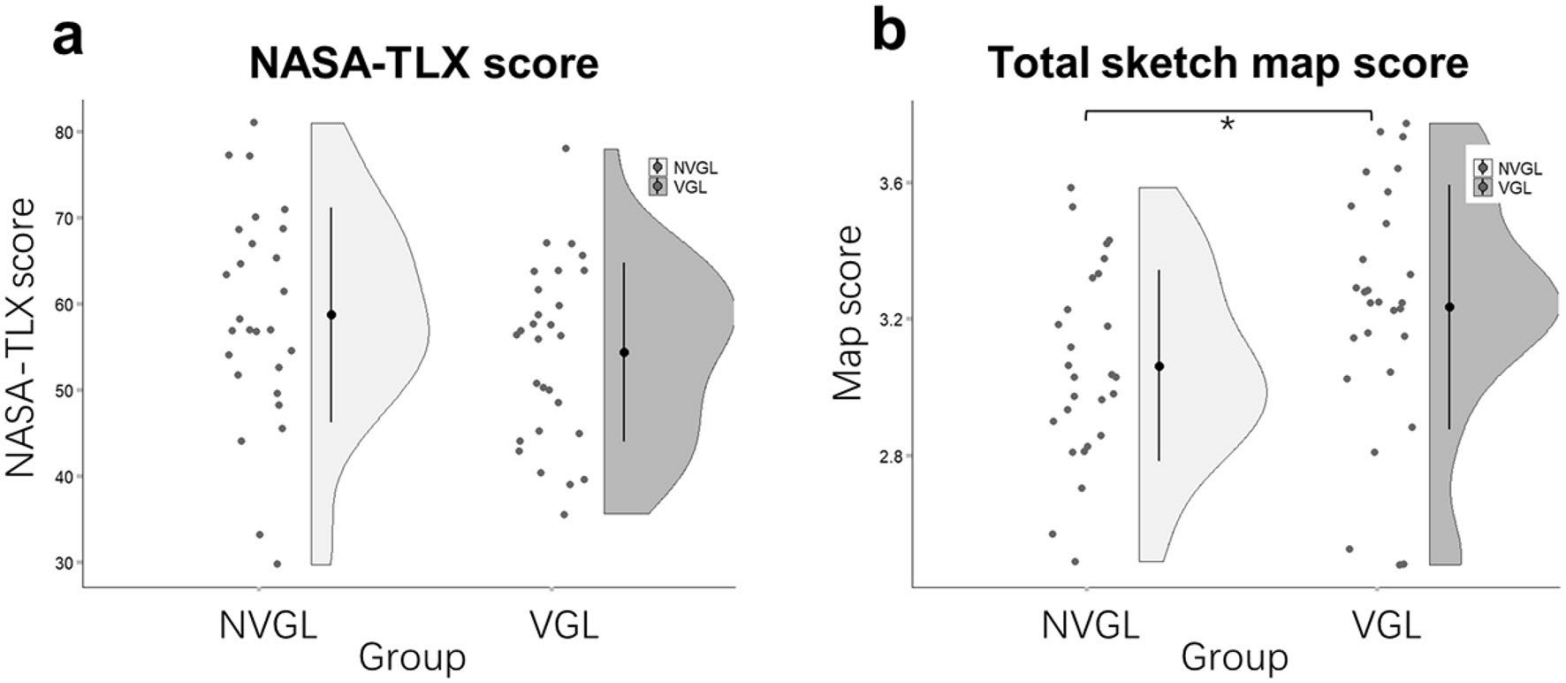
Comparisons between the VGL and NVGL groups in the map drawing task. Each dot of the scatter plot indicates the average value for one participant. **(a)** NASA task load index (NASA-TLX) score for the mental demand; **(b)** total sketch map score. The black dots represent means, and the error bars indicate standard deviations. *: *p* < .05.

The map drawing task was our basis for assessing incidental spatial knowledge acquisition during exploration. Assessments of the sketched maps were made using Gardony Map Drawing Analyzer (GMDA)^27^. Of these, the NASA Task Load Index (NASA-TLX)^41^ indicates mental workload. The results in Fig. 3a demonstrated no statistically significant difference in mental demand scores (F_1,52_ = 1.75, *p* = 0.19, partial η^2^ = 0.03), which indicates an equal level on mental workload in two groups. However, as indicated in Fig. 3b, there was a significant difference in the total map scores (F_1,51_ = 4.60, *p* = 0.04, partial η^2^ = 0.09) with higher map scores in the VGL group (M = 3.24, SE = 0.06) than that in the NVGL group (M = 3.05, SE = 0.06) by 0.19, reflecting an overall better preformation in sketch map for VGL group.

**Figure 4:**
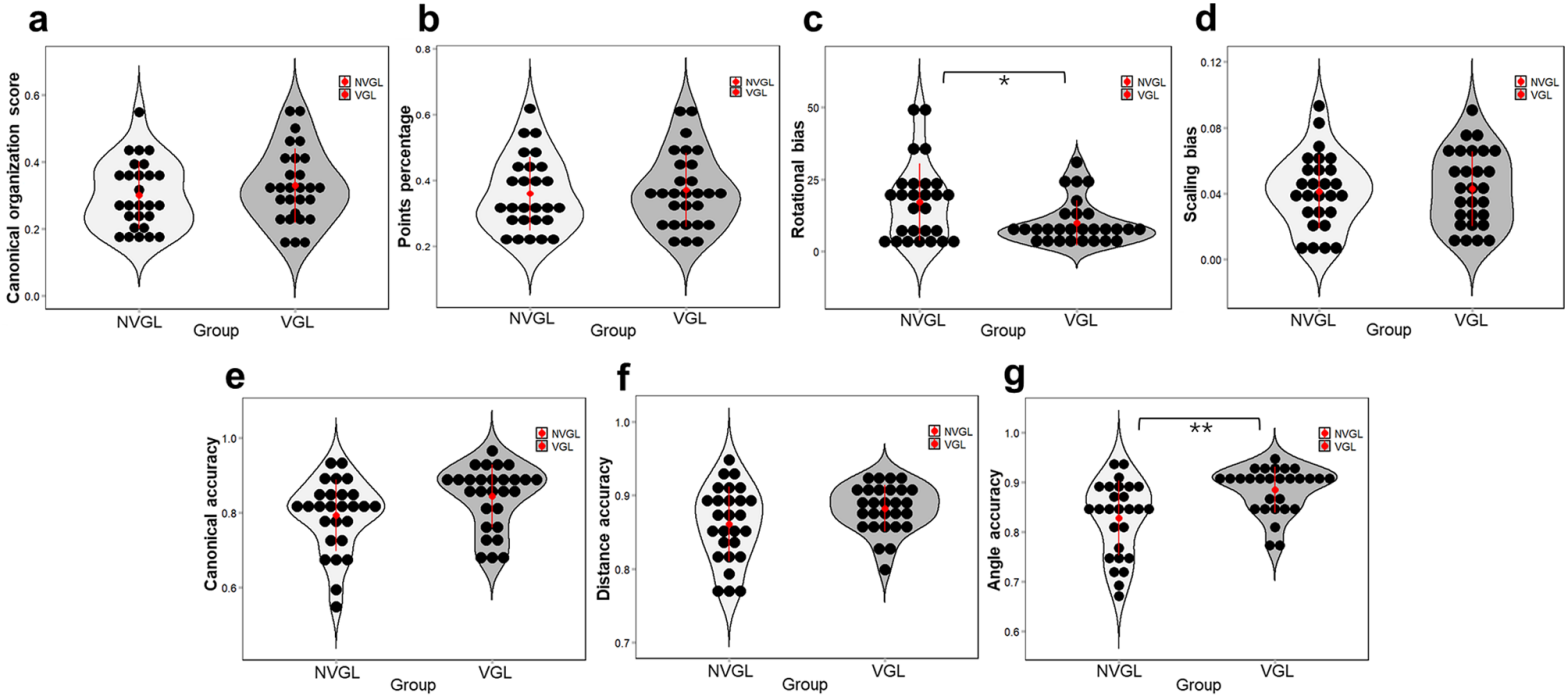
Single index scores for the VGL and NVGL groups in the map drawing task. The indexes include: **(a)** canonical organization score, **(b)** recalled points percentage, **(c)** rotational bias, **(d)** scaling bias, **(e)** canonical accuracy, **(f)** distance accuracy, and **(g)** angle accuracy. Each dot of the scatter plot indicates the average value for one participant. The black dots represent means, and the error bars indicate standard deviations. *: *p* < .05 and ***: *p* < .001.

Figure 4 charts all the single indexes used to calculate the total map scores. No significant differences between the two groups were found for canonical organization score (F_1,51_ = 1.96, *p* = 0.17, partial η^2^ = 0.04), recalled points percentage (F_1,51_ = 0.73, *p* = 0.40, partial η^2^ = 0.01), scaling bias (F_1,52_ = 0.34, *p* = 0.56, partial η^2^ = 0.007), canonical accuracy (F_1,51_ = 3.09, *p* = 0.09, partial η^2^ = 0.06) and distance accuracy (F_1,51_ = 2.77, *p* = 0.10, partial η^2^ = 0.05). However, significant differences were found for rotational bias (F_1,52_ = 4.15, *p* = 0.047, partial η^2^ = 0.07) with 6.78 degree lower in the VGL group (M = 10.08, SE = 2.20) than that in the NVGL group (M = 16.86, SE = 2.25). As the rotational bias measures the direction of angular error of inter landmark angles^27^, the significant lower rotational bias for VGL group suggests a higher precision on landmark rotation. This outperformance on angle representation of VGL group is also further shown by a significant higher angle accuracy (F_1,51_ = 7.71, *p* = 0.008, partial η^2^ = 0.13) with 0.05 percentage higher in the VGL group (M = 0.88, SE = 0.01) than that in the NVGL group (M = 0.83, SE = 0.01).

### VGL system users saw ERSP desynchronization in the theta and alpha bands of occipital-parietal clusters

**Figure 5:**
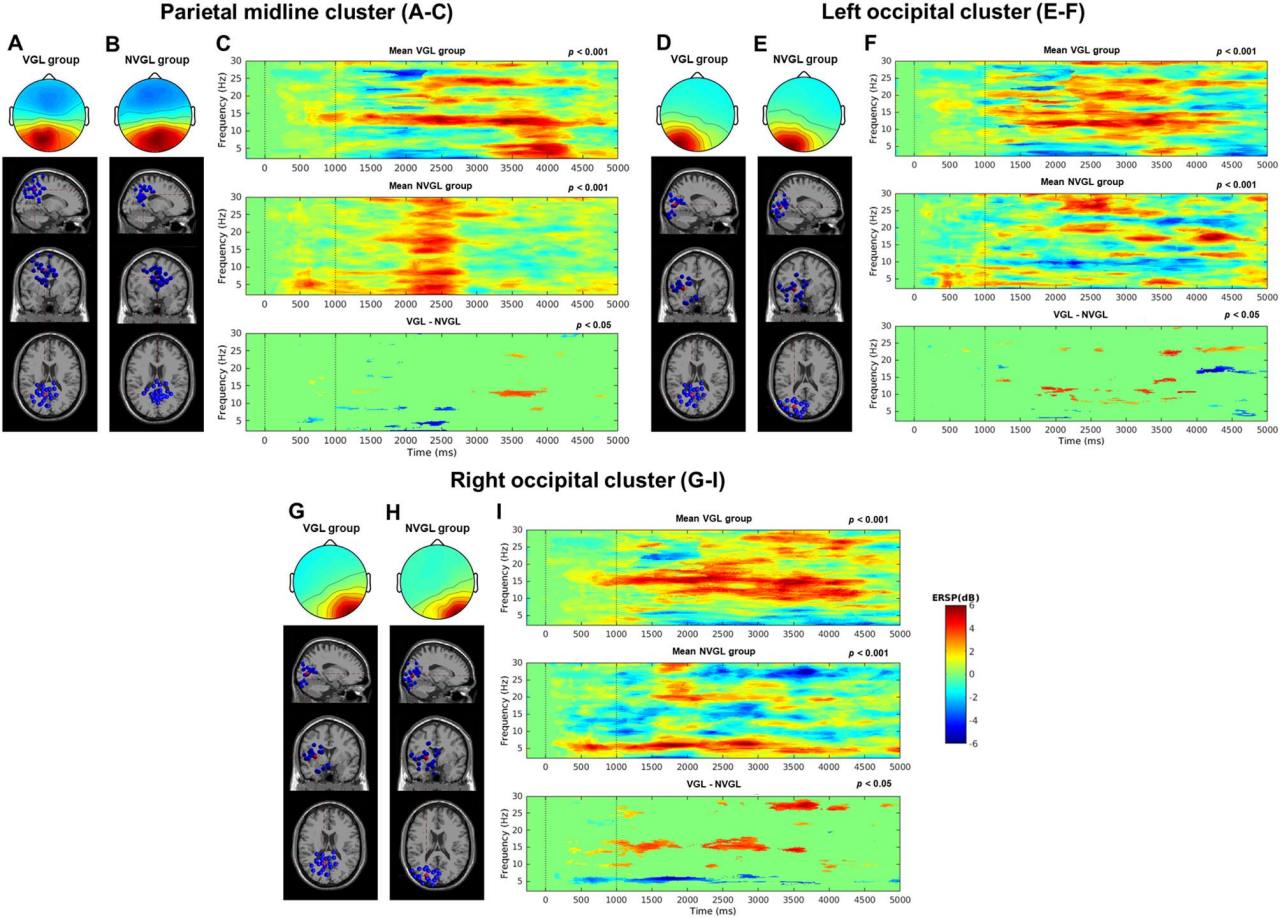
Parietal midline cluster (MNI coordinates: VGL group, *x* = 15, *y* = −53, and *z* = 33; NVGL group, *x* = 7, *y* = −26, and *z* = 45), left occipital cluster (MNI coordinates: VGL group, *x* = −22, *y* = −58, and *z* = 17; NVGL group, *x* = −11, *y* = −99, and *z* = −3) and right occipital cluster (MNI coordinates: VGL group, *x* = 15, *y* = −87, and *z* = −15; NVGL group, *x* = 29, *y* = −91, and *z* = −8). **(a,b,d,e,g,h)** Scalp maps and equivalent dipole locations of independent components at the sagittal, coronal, and top view for VGL group (**a,d,g**) and NVGL group (**b,e,h**) in parietal midline cluster (**a,b**), left occipital cluster (**d,e**) and right occipital cluster (**g,h**). (**c,f,i**) ERSPs in VGL and NVGL groups, as well as significant differences between two group conditions (ERSPs of VGL group minus the ERSPs of NVGL group) with *p* < .05 from parietal midline (**c**), left occipital (**f**) and right occipital (**i**) clusters (first dotted lines at the 0 ms time point signify the onset of a trial, and the second dotted lines at the 1000 ms time point signify the onset of stimuli). For all ERSPs, non-significant points were masked with zero values in the mean ERSPs and are displayed in green. Significant differences with respect to baseline activity are displayed in red and blue for positive and negative deviations from the baseline activity, respectively.

Figure 5 shows the spectral fluctuations in the left and right occipital clusters and parietal midline clusters as measured via ERSPs. The readings are from −1 to 5 s, where the time point 0 indicates where the participant reached one of the virtual checkpoints (see the checkpoint map in Fig. 9 of the “Materials and methods” section). The time point 1000 ms indicates where a fixation on one kind of landmark started, and the time point 5000 ms shows where the participant reached the next position (offset in ERSPs). Only one landmark of any kind was viewed before reaching the next position (offset) (i.e., a virtual global landmark in the VGL group, a local landmark in the NVGL group). For both the occipital and parietal clusters, the non-green pixels indicate differences in the ERSPs (Fig. 5c,f,i). Within one group, the significant differences level for mean ERSP is *p* < 0.001. Between the two groups, these differences were statistically significant at *p* < 0.05.

### Average ERSPs in the parietal midline cluster

The parietal midline cluster comprised 24 independent components from 18 participants for the VGL group, and 33 independent components from 19 participants for the NVGL group. The average scalp maps for these clusters along with the dipole locations are presented in Fig. 5a for the VGL group and Fig. 5b for the NVGL group. As presented in Fig. 5c, the average ERSPs for the VGL group revealed significant decreases in theta activity shortly after the onset of stimuli, and an increase in alpha power band activity, *p* < 0.001. By contrast, the ERSPs for the NVGL trials showed significant increases in the same frequency bands, *p* < 0.001. The difference in ERSPs between group conditions had statistical significance in these activity bands, *p* < 0.05, and demonstrates a pronounced suppression in theta activity and an increase in the alpha band of the VGL group compared to the NVGL group.

### Average ERSPs in the left and right occipital clusters

The left occipital cluster included 21 independent components from 16 participants for the VGL group, and 21 independent components from 15 participants for the NVGL group. The right occipital cluster included 33 independent components from 25 participants for the VGL group, and 15 independent components from 13 participants for the NVGL group. The average scalp maps for these clusters along with the dipole locations are presented in Fig. 5d and g for the VGL group and Fig. 5e and h for the NVGL group.

As shown in Fig. 5f and i, in both the left and right cerebrum, the average ERSPs in the occipital cortex for the VGL group reveals a strong increase in alpha power band activity at the onset of the landmark stimuli (*p* < 0.001). The increase became stronger for a short time after viewing the virtual global landmark. The stimulus induced theta-power suppression was also apparent. In contrast, for the NVGL group, power increased significantly in the theta bands and decreased in the alpha band (*p* < 0.001). Theta activity was increased strongly around the stimuli of local landmarks, tapering back to slight after a while. The differences in ERSPs between conditions was statistically significant for the theta and alpha bands, *p* < 0.05, and showed a significant suppression of theta activity and an increase in alpha band activity for the VGL group compared to the NVGL group.

## Discussion

In this work, we seek to explore the particular impact of the VGL system on spatial knowledge acquisition. To investigate this, we undertook the neural measures as well as the behavioral traits, most importantly, through a map drawing task with 55 participants. These 55 participants were divided into two groups—one equipped with the VGL system and one without—and asked to explore an unfamiliar environment. The exploration route for both groups was fixed. Once the participants completed their route, we asked them to sketch a map^48^ of the environment they had just explored. We hypothesized that those using the VGL system would absorb more spatial information, be more spatially aware of their environment, and draw their maps more accurately than those who did not have the aid of the VGL system.

The results of the map drawing task in Fig. 3 demonstrate significantly higher scores for the VGL system participants at the same levels of mental workload as the NVGL system participants. Here, mental workload for mapdrawing task was assessed through NASA-TLX^41^, which is a common subjective workload assessment tool. Consistent with our hypothesis, there was no difference between the cognitive workloads of the two groups in terms of NASA-TLX results. A possible reason for this is that the VGL group did more information processing in terms of mentally organizing and sketching their surroundings while exploring. This view is supported by the higher map scores achieved by this group. Thus, the comparable mental workload between VGL and NVGL group could imply that the VGL system did not carry more or even help to ease the workload of spatial memory though more information processing required comparing without VGL system.

Sketching maps is a common method of evaluating spatial knowledge gained from an environment and one that is robust to cognitive impacts^25–27^ as well as spatial memory^49,50^. The results of all the measured indexes pinpointed the main significant differences for the total map scores came from the indexes to mark the angular accuracy level of the sketch map, including angle accuracy and rotational bias. As for the measures to assess the organization related scores, including canonical organization score itself, points percentage to show successfully recalled landmarks and intersections as well as scaling bias and distance accuracy to show organization of interlandmark distances, participants in the environment with VGL and local landmarks showed an equal performance level to those in the environment with local landmarks only. This might be because, as a conspicuous and easy-to-pinpoint reference, global landmarks can help participants build a clearer directional relationship between themselves and a constant point of reference^18,19^. Thus, as a persistent reference for global landmark, VGL system could support participants to keep well-marking the rotational relationship among environment features by a clearer directional sense. This is why the participants aided by the VGL system were able to gain a more accurate performance on angular measures of incidental spatial knowledge in this new environment.

Further, because participants were not aware they would subsequently be required to draw a map, we can be sure they were retrieving spatial information from a mental memory map during the map drawing exercise. Notably, they formed this map from only one exposure to the route and environment. Still, participants from the VGL group drew much more precise maps, which indicates that the global landmarks helped them to process the environment’s orientation and structures more accurately.

Comparing the other behaviors of the two groups, the participants using the VGL system spent significantly more time in the exploration phase, as shown in Fig. 2a. This could simply because the VGL system participants hesitated during exploration, or which is more possible that they walked more slowly and were more willing to look around and attracted attention to their surroundings rather than simply following the instructions they were given. The blink interval readings, given in Fig. 2b, support this idea. A higher blink rate can be interpreted as a function of split attention^46,47^. In other words, when participants show increased interest in stimuli, the eyes tend to fixate for longer and the blink count decreases. Figure 2b reveals a significant increase in blink interval for the VGL group, which means the participants fixated for longer on their surroundings compared to the group that was not using the VGL system.

Consistent with our behavioral findings, desynchronizations in the theta and alpha power bands of the occipital and parietal sites also suggested an increased attention in visual stimuli in VGL group versus the NVGL group during guided exploration. As indicated in Fig. 5, there were significant differences in the occipital and parietal ERSPs of the VGL group versus the NVGL group in terms of theta oscillation suppression and increased alpha oscillations. We interpret the increased alpha and decreased theta power over these sites as the participants suppressing distractions from their visual system^42–45^. Previous studies on short-term memory also found alpha activity could be increased while brain was carrying more memory load^42,51^. In other words, the suppression of theta activity and the increase of alpha activity signified that more attention was being paid during neural processing. Thus, as the ERSP data for the VGL group shows, our findings with the brain dynamics are consistent with our findings for the behavior results, both demonstrating that participants using the VGL system paid more attention to spatial knowledge acquisition during the exploration phase.

In summary, the study reveals a significant improvement in incidental spatial knowledge acquisition when using the VGL system as reflected in the assessment of sketch maps. As a reference point, the VGL system encourages users to be more actively aware of their surroundings and process their environment. By enhancing user perceptions of navigation information, the VGL system can augment the navigation capabilities of users, which could be the scientific basis for a new generation of future navigation assistance systems that not only show users the way to go but also support spatial learning. In our future work, we will continue studying VGL’s effect on the efficacy of spatial learning with more physical task, e.g., assessing orientation accuracy and efficiency with VGL after a guided exploration.

## Materials and methods

### Participants

The experiment involved 55 participants: 24 females and 31 males (see Table 1 for demographic information). Experiments were conducted in the UTS Tech Lab. Before participating in the study, the experimental procedure was explained, and all participants provided informed consent. The Human Research Ethics Committee (HREC) of University of Technology Sydney (UTS) also reviewed the protocols and issued their approval (grant number: UTS HREC REF NO. ETH17-2095). All experiments were performed in accordance with relevant guidelines and regulations. None of the participants reported a history of any psychological disorders that could have affected the experimental results. To control for individual differences in spatial abilities, we administered a SBSOD test^39^ and a PTSOT test^40^ prior to conducting the experiment.

**Table 1:** Participant demographics and average orienting ability test scores at the initial testing.

Group	Women/men	Age (years)	SBSOD	PTSOT
VGL	15/13	27.57(± 4.78)	0.66(± 0.15)	37.51(± 26.49)
NVGL	9/18	28.19(± 5.33)	0.67(± 0.17)	26.31(± 17.11)

Standard deviations are shown in parentheses.

*SBSOD* Santa Barbara Sense of Direction scale, *PTSOT* Perspective Taking/Spatial Orientation Task.

### The VGL system setup

The virtual global landmarks were displayed as transparent, two-dimensional silhouettes of the real landmark in the VR scenario, which serve as a stable reference for the direction of specific locations without disturbing the overall environment. With the displaying of these virtual landmarks, users were encouraged to continuously compute directions from a particular location^52^. The silhouettes were presented in the direction of the global landmark within the participant’s sightline. Whether walking or turning, as long as the participant looked in that direction, they were able to see either see the real landmark or the silhouette if it was blocked by another object, as shown in Fig. 1. During their trials, the participants were shown one of three virtual global landmarks: a lighthouse, the Sydney Opera House, or the Sydney Tower Eye.

### VR and EEG setup

**Figure 6:**
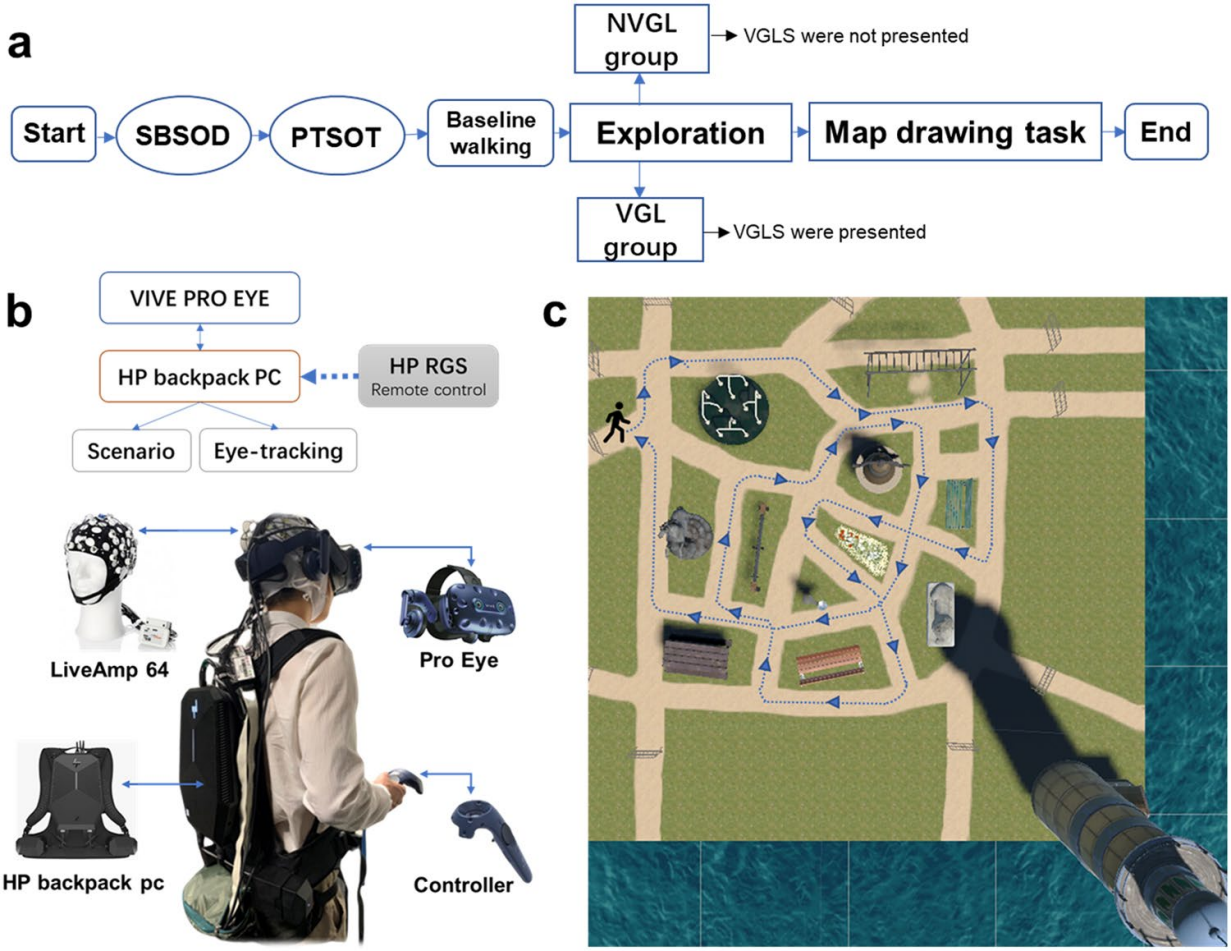
Experiment procedure. **(a)** Overview of the experimental procedure design. First, the participants completed a Santa Barbara Sense of Direction (SBSOD) questionnaire about their sense of direction and a Perspective Taking/Spatial Orientation Task (PTSOT), which assesses spatial orienting ability. They were then asked to walk in a square meadow VR environment. Next, participants from NVGL and VGL group started walking through Sydney Park along a pre-defined route by auditory instructions without and with VGL system respectively. Last, the map drawing task was conducted. **(b)** The gear setup for each participant. During the tasks in the VR environment, participants were wearing a 64-channel EEG cap covered by the VR headset with an HP backpack PC on their back and were holding a controller. The pro eye headset was connected to the HP backpack PC with scenario running inside. The remote-control software (HP Remote Graphics Software, RGS) was applied in another PC to monitor the scenario and related activity. **(c)** A pre-defined route map of exploration. Auditory instructions were used to guide participants through the route. The background map is a top view of the “Sydney Park” scenario. The plants and trees inside and surrounding the scenario were removed for a clear view of the path.

Figure 6b provides an overview of the setup for participants. The Sydney Park scenario was based on VR but imitates the real environment of the Sydney Botanical Gardens. The scenario was fully immersive so as to hold participants’ attention during the full duration of the navigation experiments. We used HTC’s Vive Pro eye headset with an embedded Tobii eye tracker. The Vive Pro eye uses a dual OLED 3.5" diagonal display with a resolution of 1440 × 1600 pixels per eye (2880 × 1600 pixels combined) and a refresh rate of 90 Hz, as reported by HTC. The participant’s head position was principally tracked with embedded inertial measurement units, while an external lighthouse tracking system cleared the common tracking drift with a 60 Hz update rate. We tracked the eye activity of participants using the Tobii eye tracker at a sampling rate of 120 Hz.

The EEG data were recorded continuously using Brain Vision’s LiveAmp 64 system (Brain Products, Gilching, Germany) using 64 active electrodes mounted on an elastic cap. The sampling rate was 500 Hz with a low-pass filter of 131 Hz. The electrodes were positioned according to an extended 10–20 system^53^. The EEG signals were referenced to the electrode located at FCz and the impedance of all sensors was kept below 5kΩ. EEG events were created when the participants’ fixated on the surface of a defined landmark, both real and virtual. All data streams from the EEG cap, eye tracker and head-mounted display were synchronized with Lab Streaming Layer (LSL).

**Figure 7:**
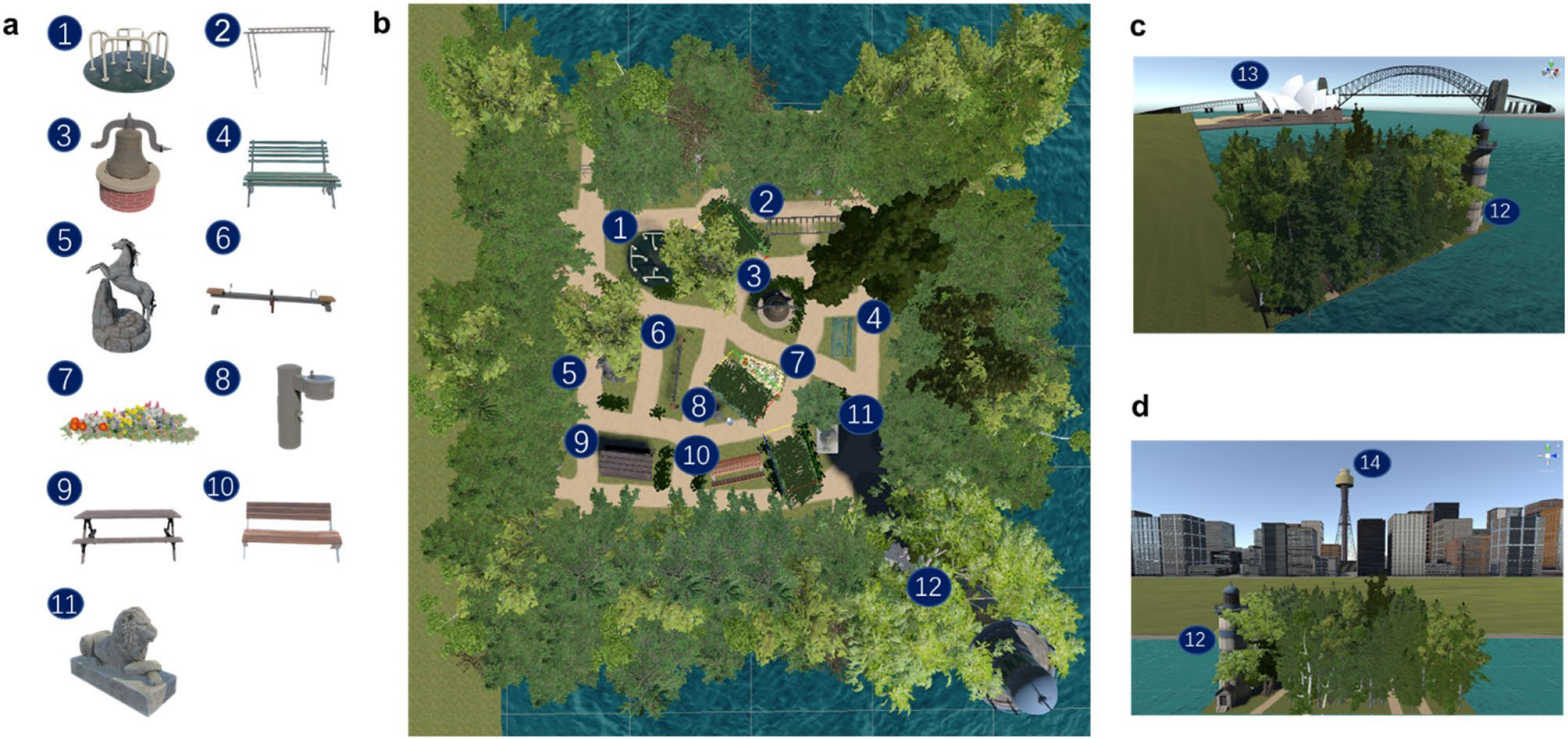
VR scenario of Sydney Park. **(a)** Map of local landmarks in the scenario. Label 1 to Label 11 represent a spinning wheel, monkey bars, a bell sculpture, a green bench, a horse sculpture, a seesaw, a parterre, a water fountain, a picnic table, a brown bench and a lion sculpture, respectively. **(b)** A birds-eye view of the scenario. Label 12 represents a lighthouse. The remaining landmark label beside each landmark is consistent with the respective label shown in **(a)**. **(c)** Sydney Opera House side view of scenario. **(d)** Sydney Tower Eye side view of scenario. This scenario was developed in Unity 2018.3.5f1 (Unity Technologies, USA, https://unity.com/).

Sydney Park was created in Unity 2018.3.5f1 (Unity Technologies, USA). Figure 7 presents the screen views of Sydney Park scenario in Unity. The Sydney Park environment consists of eleven local landmarks (Label 1 to Label 11 in Fig. 7a,b) and three global landmarks (Label 12 to Label 14 in Fig. 7b–d, including a lighthouse, the Sydney Opera House and the Sydney Tower Eye), in combination with paths, intersections, bushes, trees, etc. Two sides of the scenario were extended with only the sea, and a lighthouse standing in the corner next to the ocean, as shown in Fig. 7b. Of the remaining two sides, one had a view of the Sydney Opera House along with Sydney Harbour Bridge behind (Fig. 7c) and the other had a view of the Sydney Tower Eye with other tall buildings as a city skyline (Fig. 7d). The background views are similar to the actual views from the Royal Botanical Gardens, whereas the inner layout of Sydney Park is unique. To control the visibility proportion of all global landmarks, we used background plants, e.g., trees, to ensure global landmarks were obscured. The visible status for all global landmarks was checked at each checkpoint (checkpoint map is shown in Fig. 9). Overall, global landmarks were blocked from view for nearly 60% of the time during the experiment.

### Experiment procedure

An overview of the experimental procedure is shown in Fig. 6a.

#### Pre-test

Before the participants performed the exploratory and navigational tasks, we conducted a pre-test (SBSOD^39^, PTSOT^40^) to assess their individual spatial abilities. The participants were not aware of the experimental procedure to come while completing this phase. Every participant completed the SBSOD questionnaire as a subjective measure of his or her sense of direction, as well as a PTSOT to evaluate their spatial orientation ability.

### Exploration phase

All participants were randomly divided into two groups in this phase, one group explored Sydney Park along with VGL system (VGL group), the other without (NVGL group). Each participant first had five minutes to walk inside a meadow area in the VR environment. Participants were given this time to explore until feel comfortable to do physical movement in the VR environment. If any uncomfortable feedback occurs, the experiment would be terminated. In the next step, participants were given an instruction based on the map of Royal Botanical Gardens and its surroundings to explain the background layout of our Sydney Park scenario as well as the global landmarks. For VGL group, during instruction about global landmarks, VGL was also explained. Except the global landmarks within the background surrounding, participants were not shown any local landmarks as well as the scenario’s inner layout. After the instruction, scenario was presented in VR and participants started walking through the Sydney Park scenario along a fixed, predefined route with the assistance of auditory instructions (see Fig. 6c and Movie S1 in the supplementary). Turn-by-turn auditory instructions were used to build a familiar navigation aid of automatic orientation environment. To control all participants from both groups travelling the same fixed route, once wrong response to the verbal prompts occurred, the instructor who monitored the experiment would immediately ask the participants to stop and instruct them back to the right direction before reaching a different path. This was intended to standardize how participants explored the environment, and in the meantime, the use of auditory instruction could help to observe and investigate the VGL system’ effects on spatial learning in a common guided-navigation environment. All target landmarks involved in the following tasks were passed just twice while navigating the fixed route.

### Map drawing task and NASA-TLX questionnaire

After the exploration, each participant was given a blank 11.7 × 16.5-inch sheet of paper, a pencil and an eraser. They were then instructed to draw any information they remembered about the scenario, including landmarks, paths, etc., They were not allowed to conduct the scenario or listen to the instructions again. Following the map drawing task, each participant filled out a NASA Task Load Index questionnaire (NASA-TLX)^41^.

### Sketch map analysis

**Figure 8:**
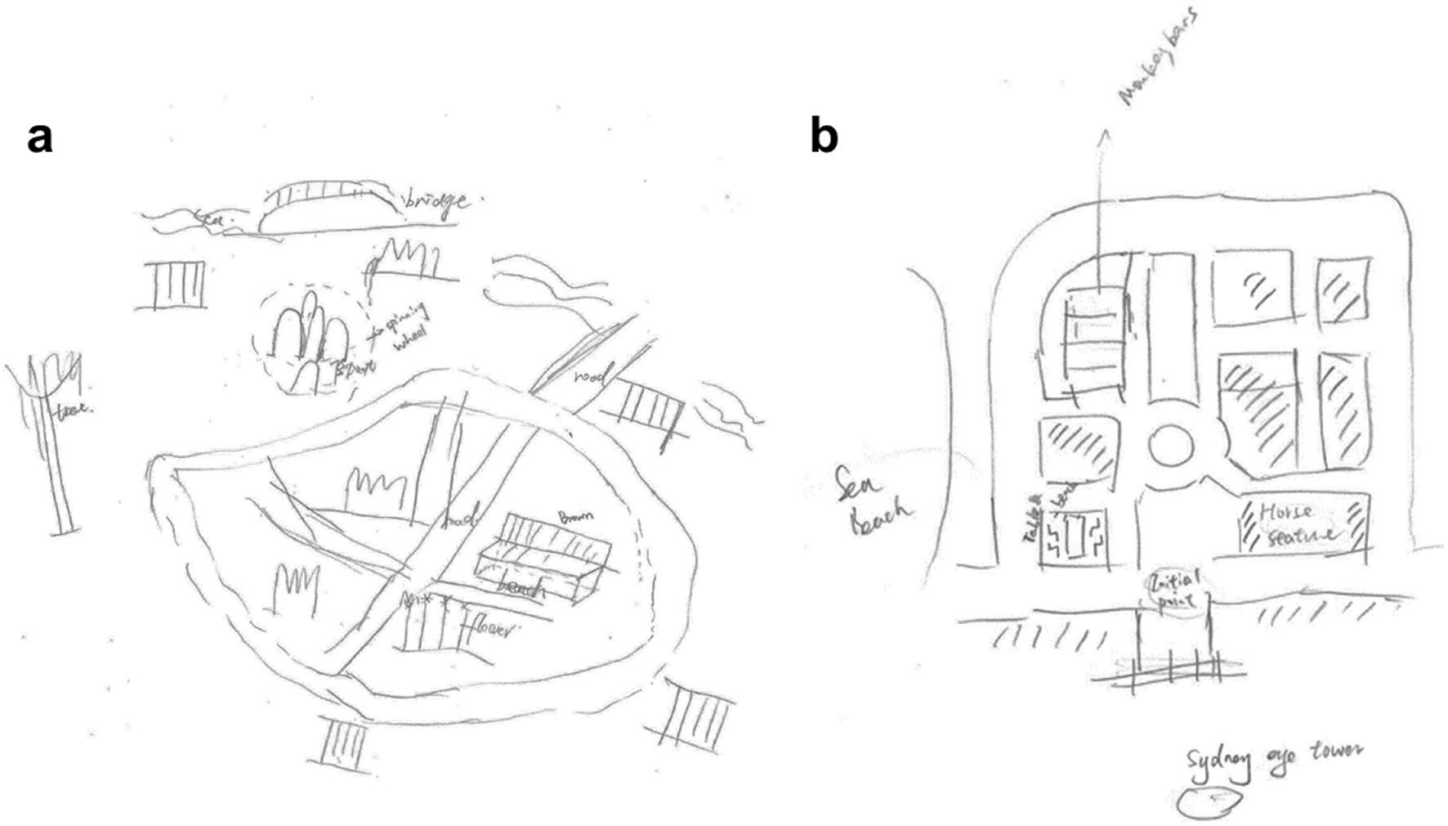
Sketch maps from participants. **(a)** An example of sketch maps from NVGL group. **(b)** An example 453 of sketch maps from VGL group.

We analyzed the sketched maps with the Gardony Map Drawing Analyzer (GMDA)^27^. This software package provides quantitative measures of map accuracy using novel measures, unique to GMDA, that rely on both pairwise comparisons and traditional bidimensional regression parameters^54^. Figure 8 show examples of sketch maps from participants. During scoring the total map score for each sketch map, we drew upon all the critical measures provided by GMDA, including canonical organization (with a square-root-corrected measure), canonical accuracy, points percentage, rotational and scaling biases, distance accuracy, and angle accuracy. The total map score can be represented by an equation as follows: :

1 $$\begin{aligned}\text{Total map score} &= \text{SQRT} \left(\text{canonical organization}\right)+\text{canonical accuracy} + \text{ points percentage}\\ &\quad- \frac{\text{rotational bias}}{360}-\text{ scaling bias}+\text{ distance accuracy }+\text{ angle accuracy}\end{aligned}$$

The total map score ranges from −2 to 5 and a higher score indicates an overall better preformation in sketch map. For all the single measures, the canonical organization measures the overall configuration accuracy of the sketch map, with penalties for missing landmarks while canonical accuracy measures the configurational accuracy of only the drawn landmarks. These two measures are all range from 0 to 1 with higher scores indicating better configural accuracy and landmark recall. The points percentage records the successfully recalled points of the explored environment, including landmarks and intersections, as percentages, which ranges from 0 to 1 with higher scores indicating higher proportion of successful recalled points. The scaling bias measures the direction of scaling of the inter landmark distances, which ranges from 0 to 1 with lower scores indicating higher precision on landmark scaling. Rotational bias measures the direction of angular error of inter landmark angles, which ranges from 0 to 360 with lower scores indicating higher precision on landmark rotation; however, to equal this index to others, we took $\frac{\mathrm{rotational bias}}{360}$ instead as shown in Eq. (1) to make it range from 0 to 1 as well. Distance and angle accuracy reflect the accuracy of inter landmark distances and angles, which all range from 0 to 1 with higher scores indicating more accurate interlandmark distance and angle representation, respectively.

### EEG analysis

#### Pre-processing

All raw EEG data were imported into MATLAB version 2018a (MathWorks Inc., USA) for processing. We used the EEGLAB toolbox version 2020.0^55^ to aid in the analysis. For each participant’s raw data, we first checked the data quality by eye to ensure the EEG data was consistently recording with active movements during experiment. Of the 55 participants, data of one participant from NVGL group was excluded due to poor EEG quality. The raw data for the remaining 54 participants were first bandpass filtered from 1 to 100 Hz and downsampled to 250 Hz. Then, data from each single task were merged into one large EEG dataset for the following pre-processing steps. Line noise and flatlines were removed in turn using the *cleanline* and *clean_flatlines* functions in EEGLAB. Noisy channels were rejected with the *clean_channels* function. All missing EEG channels were interpolated by spherical splines before re-referencing to the average of all channels. Noisy data in the time domain were removed through automatic continuous data cleaning. On average, 48.55% ± 17.79% of the data in the time domain were removed. The data were then submitted to adaptive mixed independent component analysis to obtain independent components^55,56^. The equivalent dipole model of each independent component was computed using a boundary element head model as implemented in EEGLAB’s DIFIT2 routines^57^. Last, the sphere and weights of the ICA and dipole models were copied back to the pre-processed but uncleaned EEG single-task data for further analysis. (There was no cleaning in the time domain).

#### Event-related spectral perturbation (ERSP)

**Figure 9:**
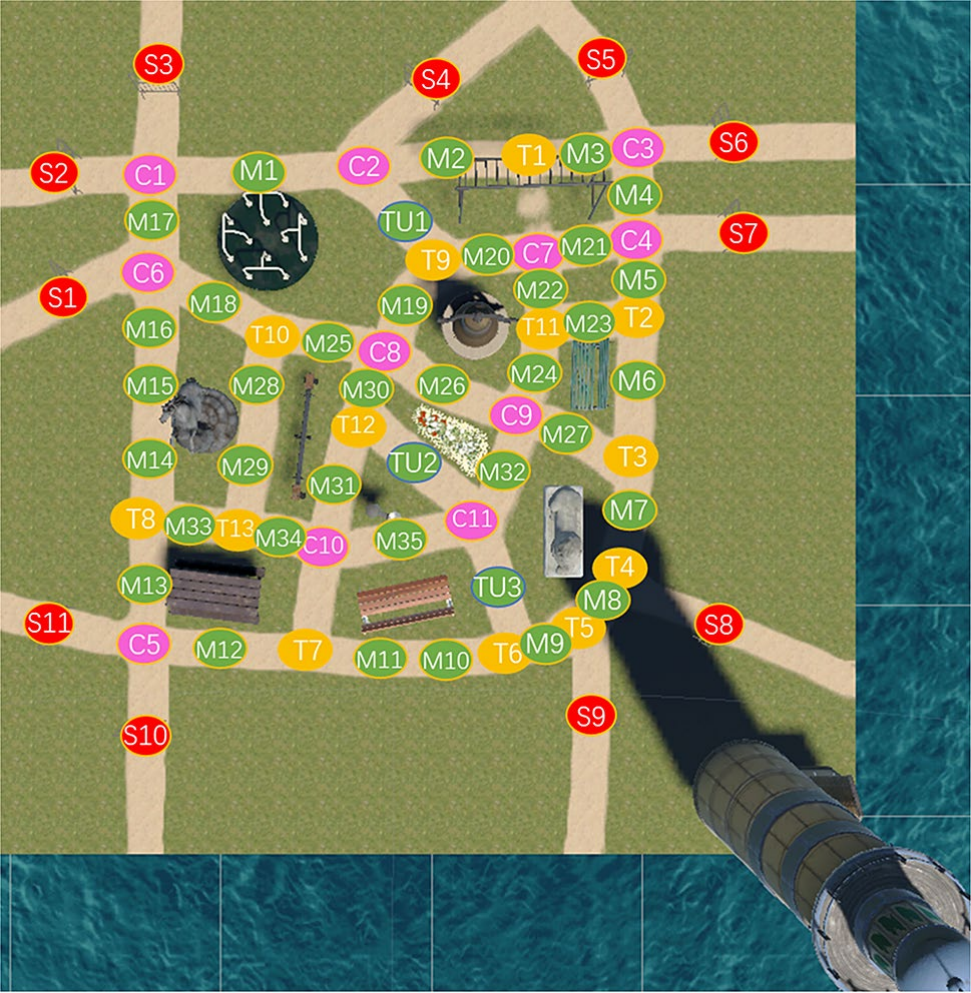
Checkpoint map. The red checkpoints define the starting points; the green checkpoints are those in the middle of one path; the purple checkpoints appear at crossroads; and the yellow checkpoints represent a T intersection. The background map is a top view of the “Sydney Park” scenario.

The cleaned data for the exploration was extracted with a time window of [−1 s to 7 s]. The onset and offset events were generated based on checkpoints (see Fig. 9). For each single fixation period between two checkpoints (on the virtual global landmarks in the VGL group or the local landmarks in the NVGL group), the onset event was generated at the first checkpoint, and an offset event was defined at the second checkpoint. Bad epochs were detected and removed based on component activities using the *autorej* function. On average, 0.08 ± 0.40% epochs of the NVGL group condition were removed; 6.95 ± 2.89% epochs of the NVGL group condition were removed. All trials were then time-warped to time lengths of [−1 s to 6 s], and ERSPs were plotted for each independent component with the *newtimef* function.

#### Independent component clustering

The independent components from all participants were first selected with less than 15% residual variation of the equivalent dipole model, and the components with an equivalent dipole model located outside the head sphere were removed. Then, the selected components were clustered using K-means clustering in EEGLAB. To avoid the “double dipping” problem^58,59^, only dipole locations were included as the measure for clustering. For the ERSP analysis, we focused on the clusters of components located in or near the parietal cortex and occipital cortex. We looked for active neural dynamic differences related to spatial navigation^34,60–63^ or visual stimuli^42,44^ between the VGL and NVGL groups. We used the Talairach client tool^64,65^ to evaluate the nearest gray matter of the dipole locations from the targeted cluster centroid and clustered components.

#### Group-level ERSPs and statistics

We first computed the ERSPs at the single independent component level based on the cluster of interest, then averaged them at the participant level, and finally at the group level. The time–frequency data of all independent components from the same participant were averaged. Then, the ERSPs of all participants were averaged for the final ERSPs at the group level. Significant differences from the baseline activity are displayed in red for positive deviations, blue for negative deviations, and green for nonsignificant differences. We determined conditional differences using the *newtimef* function with a statistical threshold of *p* < 0.001 for all selected independent components. A global overview of our processing steps for the EEG analysis is provided in Fig. S2 in the Supplementary.

### Statistical analysis

The statistical analyses were conducted using SPSS Statistics 26 (IBM Analytics, Armonk, USA). Data visualizations were created with the *ggplot* function of R^66^ (RStudio Inc, USA). We computed one-way ANOVAs for the between-subject factor trial by group (VGL and NVGL) for the exploration, as well as for the map drawing task and for eye activity. The dependent values for exploration analysis were the response time (time spent in exploration) and blink intervals. For the map drawing task, the dependent values included the NASA-TLX score and total sketch map score along with all single indexes, i.e., canonical organization, recalled points percentage, rotational bias, scaling bias, canonical accuracy, distance accuracy and angle accuracy. Each measure was calculated separately for the VGL and NVGL group.

For all measures, we first explored the data to check if outliers existed for the within-subject factor computations. All outliers inspected by boxplots for values greater than 1.5 box lengths from the edge of the box were removed. In addition, we ran a Shapiro–Wilk test to determine whether the mean values for both groups were normally distributed. We used Spearman's rank-order correlation to assess the relationship between individual spatial ability factors (SBSOD and PTSOT scores) and all measures. The Spearman’s correlations result between each individual’s spatial ability and the measures are shown in the Supplementary Fig. S1. With the significantly correlated factors, we then used the scores from these tests as covariates to assess how much the participants’ inherent, subjective sense of direction and orientating ability affected their completion of the navigation tasks.

## Supplementary Information

Supplementary Information.

Supplementary Video S1.
