# Supplementary material for: Using virtual global landmark to improve incidental spatial learning
Source: Sci Rep. 2022 Apr 25;12:6744. doi: 10.1038/s41598-022-10855-z (PMC9039035; doi:10.1038/s41598-022-10855-z)
Supplement: Supplementary file 1 — Supplementary Information. [file 41598_2022_10855_MOESM1_ESM.docx]

# Supplementary to the manuscript:

# Using Virtual Global Landmark to Improve Incidental Spatial Learning

Jia Liu, Avinash Kumar Singh and Chin-Teng Lin

Australian Artificial Intelligence Institute, School of Computer Science, Faculty of Engineering and Information Technology, University of Technology Sydney, Australia

This supplementary material supports the information presented in the manuscript. The additional evidence provided here includes:

a. Correlation results of the individual spatial ability factors (SBSOD and PTSOT scores) and the dependent measures

# b. The effects results of gender as the between-subjects factor of ANOVAs

c. The EEG processing pipeline for the EEG analysis section

d. Video of the two groups performing the exploration

# Correlation results of the individual spatial ability factors and dependent measures

To control for individual difference in spatial ability, we used Spearman's rank-order correlations to assess the relationships between the individual spatial ability factors (SBSOD and PTSOT scores) and all measures. The results for each group are shown in Figure S1. With significantly correlated factors for both groups, we used the individual spatial ability factors as a covariate to assess how much the participants’ inherent, subjective sense of direction and orientating ability affected their completion of the tasks.


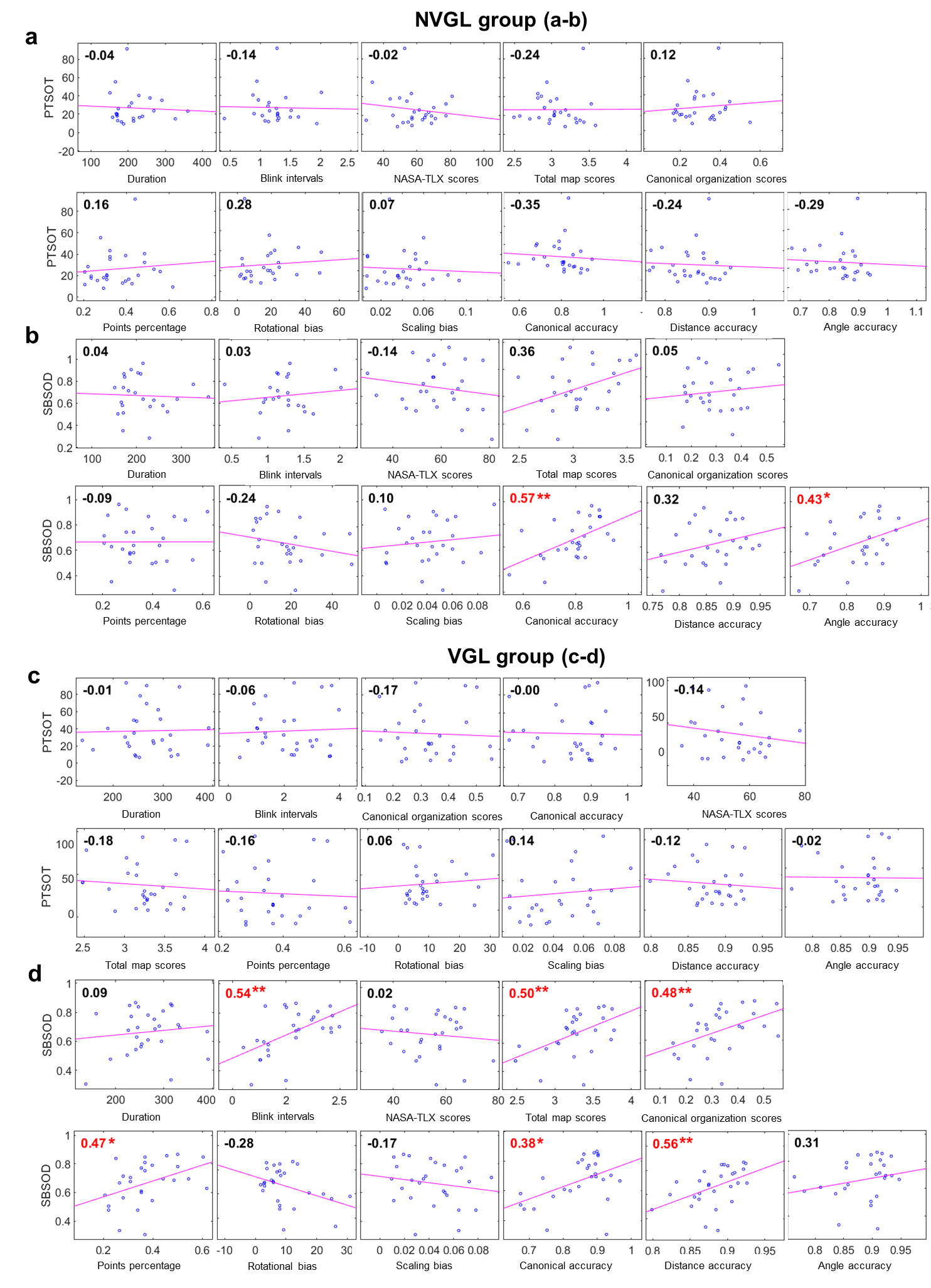


**Figure S1: Correlation result of individual spatial ability factors and dependent measures.** (a-b) Measures correlated with PTSOT (a) and SBSOD (b) for NVGL group respectively. (c-d) Measures correlated with PTSOT (c) and SBSOD (d) for VGL group, respectively. Each figure shows the computed correlation coefficient with the number in the top left corner (red color indicated for statistical significance; *, ** indicated for *p* <.05, *p* <.01, respectively.

# The effects results of gender as the between-subjects factor of ANOVAs

While analyzing one-way ANOVAs for our variables presented in results section of our manuscript, gender was added as a between-subjects factor, however, there is no significant interaction between gender and group condition (VGL and NVGL groups) for all variables. The test results of between-subjects effects are as follows:

(1) time duration in exploration: F_1,51_ = 3.19, *p* = .08, partial η^2^ = .06;

(2) average blink interval in exploration: F_1,48_ = .02, *p* = .90, partial η^2^ = .00;

(3) NASA-TLX score in mapdrawing task: F_1,51_ = .24, *p* = .63, partial η^2^ = .01;

(4) total map score in mapdrawing task: F_1,51_ = .41, *p* = .53, partial η^2^ = .01;

(5) canonical organization score in mapdrawing task: F_1,51_ = 1.20, *p* = .28, partial η^2^ = .02;

(6) recalled points percentage in mapdrawing task: F_1,51_ = .96, *p* = .33, partial η^2^ = .02;

(7) rotational bias in mapdrawing task: F_1,51_ = 1.69, *p* = 20, partial η^2^ = .03;

(8) scaling bias in mapdrawing task: F_1,51_ = 1.40, *p* = .24, partial η^2^ = .03;

(9) canonical accuracy in mapdrawing task: F_1,51_ = .01, *p* = .91, partial η^2^ = .00;

(10) distance accuracy in mapdrawing task: F_1,51_ = .01, *p* = .93, partial η^2^ = .00;

(11) angle accuracy in mapdrawing task: F_1,51_ = .15, *p* = .71, partial η^2^ = .00.

# EEG processing pipeline

The processing pipeline for EEG analysis is shown in Figure S2.


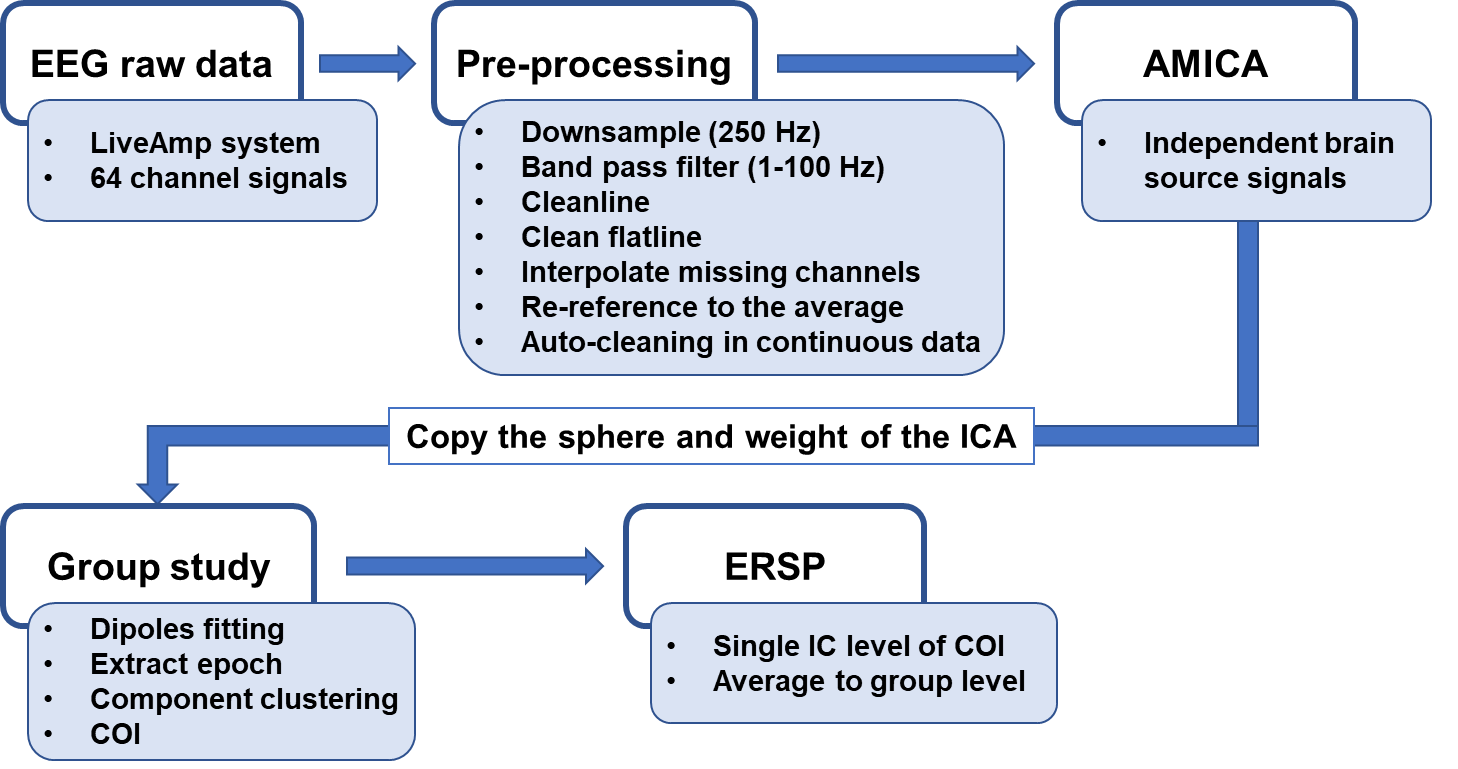


**Figure S2 EEG preprocessing pipeline**

**Video of exploration**

Video from the user’s view in our VR environment. These were recorded synchronously with the real-world video recording. This video shows how participants from two group walked through the Sydney Park scenario during the exploratory phase of the experiment, assisted by auditory instructions.
